# A Rare Case of Pediatric Infective Endocarditis Initially Presenting as Meningitis in a Previously Healthy Child

**DOI:** 10.7759/cureus.87712

**Published:** 2025-07-11

**Authors:** Tuqa A Abdulsalam, Farheen Khan, Haytham Hussein, Moza Alhammadi, Alia Magzoub

**Affiliations:** 1 General Pediatrics, Al Jalila Children's Hospital, Sharjah, ARE; 2 General Pediatrics, Al Jalila Children's Speciality Hospital, Dubai, ARE; 3 Pediatric Cardiology, Al Jalila Children's Speciality Hospital, Dubai, ARE; 4 Infectious Diseases, Al Jalila Children's Speciality Hospital, Dubai, ARE

**Keywords:** bacterial meningitis mimic, cerebritis, mitral valve vegetation, pediatric infective endocarditis, septic emboli, staphylococcus aureus, systemic embolization, transesophageal echocardiogram, valve abscess

## Abstract

Infective endocarditis (IE) is a rare but life-threatening condition in children, particularly in those without heart disease. Delayed diagnosis can lead to serious systemic complications. We report the case of an eight-year-old previously healthy girl, initially misdiagnosed with bacterial meningitis, who developed multiple septic embolic events (cerebral infarcts, digital gangrene, and splenic and renal infarction) despite being on antibiotic therapy. Repeat echocardiograms revealed mitral valve vegetation and an aortic root abscess. Blood cultures (aerobic and anaerobic) grew methicillin-sensitive *Staphylococcus aureus* (MSSA). She underwent successful surgical management via a modified Ross procedure, followed by a six-week course of intravenous (IV) flucloxacillin, resulting in full recovery. This case underscores the diagnostic challenges of pediatric IE in patients without known cardiac risk factors and highlights the critical role of serial imaging, multidisciplinary care, and early surgical intervention in achieving favorable outcomes.

## Introduction

Infective endocarditis (IE) is a rare but potentially life-threatening condition in children, with an estimated incidence of 0.05 to 0.12 per 1,000 hospital admissions [1]. Although the risk is higher among children with congenital heart disease (CHD) or indwelling central venous catheters, IE can also occur in children with structurally normal hearts. Despite advances in diagnostic techniques and surgical interventions, pediatric IE continues to be associated with significant morbidity and mortality, largely due to delayed diagnosis and a high rate of complications [2].

The clinical presentation of IE in children is highly variable and often nonspecific, which can hinder timely recognition. Fever is the most common symptom, though signs of systemic embolization, such as petechiae, focal neurologic deficits, or organ infarctions, may also be present [2,3]. *Staphylococcus aureus* has become the most frequently isolated pathogen in pediatric IE, especially among patients without predisposing heart conditions, and is associated with a more aggressive clinical course and a higher risk of embolic events [4,5].

Neurological complications, such as embolic stroke, cerebritis, and brain abscess, occur in approximately 20%-40% of pediatric IE cases, contributing to longer hospital stays and increased mortality [5]. Early neuroimaging should therefore be considered in any child with neurological signs or a persistent fever despite appropriate therapy.

Diagnosis is guided by the modified Duke criteria, which integrate microbiological and echocardiographic findings [1]. However, vegetations may be missed on initial transthoracic echocardiography (TTE), particularly in cases of deep-seated infections or early disease. In such cases, further imaging, such as TEE, may be required for confirmation [4].

Management involves prolonged IV antimicrobial therapy, typically for 4-6 weeks, tailored to the identified organism and its sensitivities. Surgical intervention is indicated in cases of structural damage, persistent infection, or complications such as abscess formation or systemic embolization [1,3]. A multidisciplinary approach is critical to optimizing outcomes in pediatric patients.

We present the case of an eight-year-old immunocompetent girl who was initially misdiagnosed with bacterial meningitis before being diagnosed with MSSA IE and systemic embolization. This case illustrates the diagnostic challenges of pediatric IE and highlights the importance of serial imaging, clinical vigilance, and early surgical intervention.

## Case presentation

A previously healthy eight-year-old girl was brought by her parents with a three-day history of fever, headache, vomiting, and reduced activity. The headache was described as intermittent, frontal in location, and worsened with fever. There was no history of photophobia, seizures, rash, upper respiratory symptoms, or sick contacts within the household. She had received two days of cefixime from a local clinic prior to this presentation.

On examination, the child appeared lethargic but not toxic. Neurologically, she was alert and oriented, with positive meningeal signs but no focal deficits. Cardiovascular examination revealed a grade 2/6 systolic murmur at the left lower sternal border, with regular capillary refill. The lungs were clear to auscultation, and no rash was noted.

Initial laboratory work-up showed a complete blood count within normal limits (Table 1).

**Table 1:** Complete blood count upon admission

Test name	Result	Reference range
White blood cell (WBC) count	8.9	5.0-13.0 × 10³/µL
Red blood cell (RBC) count	4.61	4.00-5.20 × 10⁶/µL
Hemoglobin, blood	12.0	11.5-15.5 g/dL
Hematocrit	34.9	35.0-45.0%
Mean corpuscular volume (MCV)	75.7	77.0-95.0 fL
Mean corpuscular hemoglobin (MCH)	26.0	25.0-29.0 pg
Mean corpuscular hemoglobin concentration (MCHC)	34.4	31.5-34.5 g/dL
Red cell distribution width (RDW)	13.4	11.5-14.0%
Platelet count	97	170-450 ×10³/µL
Mean platelet volume (MPV)	12.0	7.4-10.4 fL
Neutrophils, absolute	7.45	2.0-8.0 × 10³/µL
Lymphocytes, absolute	0.77	1.0-5.0 × 10³/µL
Monocytes, absolute	0.61	0.2-1.0 × 10³/µL
Eosinophils, absolute	0.00	0.10-1.0 × 10³/µL
Basophils, absolute	0.03	0.0-0.1 × 10³/µL

Elevated procalcitonin and C-reactive protein levels were noted (Table 2).

**Table 2:** Inflammatory markers upon admission

Test name	Result	Reference range
C-reactive protein	135.5	0-5 mg/L
Procalcitonin (PCT)	5.93	<0.5 ng/mL

Blood cultures grew gram-positive cocci, later identified as MSSA (Table 3).

**Table 3:** Initial blood culture upon admission

Blood culture organism	Sensitivity
Staphylococcus aureus	Oxacillin: susceptible
	Trimethoprim + sulfamethoxazole: susceptible

Three repeat blood cultures following the initial positive result were all sterile. Cerebrospinal fluid (CSF) cultures were also negative (Table 4).

**Table 4:** Comprehensive cerebrospinal fluid (CSF) analysis results

Parameter	Result	Reference range/notes
Color	Colorless	-
Appearance	Clear	Reference: clear
Coagulum	Not visible	Reference: not visible
Total nucleated cell count	205	-
White blood cell (WBC) count	204	0-7 × 10⁶/L
Red blood cell (RBC) count	0	0-5 × 10⁶/L
Mononuclear cells	4%	-
Polymorphonuclear cells	96%	-
Neutrophils	96%	Differential count
Lymphocytes	1%	Differential count
Monocytes	3%	Differential count
Cerebrospinal fluid (CSF) protein	49	15-40 mg/dL
Cerebrospinal fluid (CSF) glucose	57	60-80 mg/dL
Gram stain	Few WBCs, no organisms seen	Reference: no organisms seen
Aerobic culture	No growth	No organisms after 48 hours
Anaerobic culture	No growth	No anaerobic organisms after 5 days

**Figure 1:**
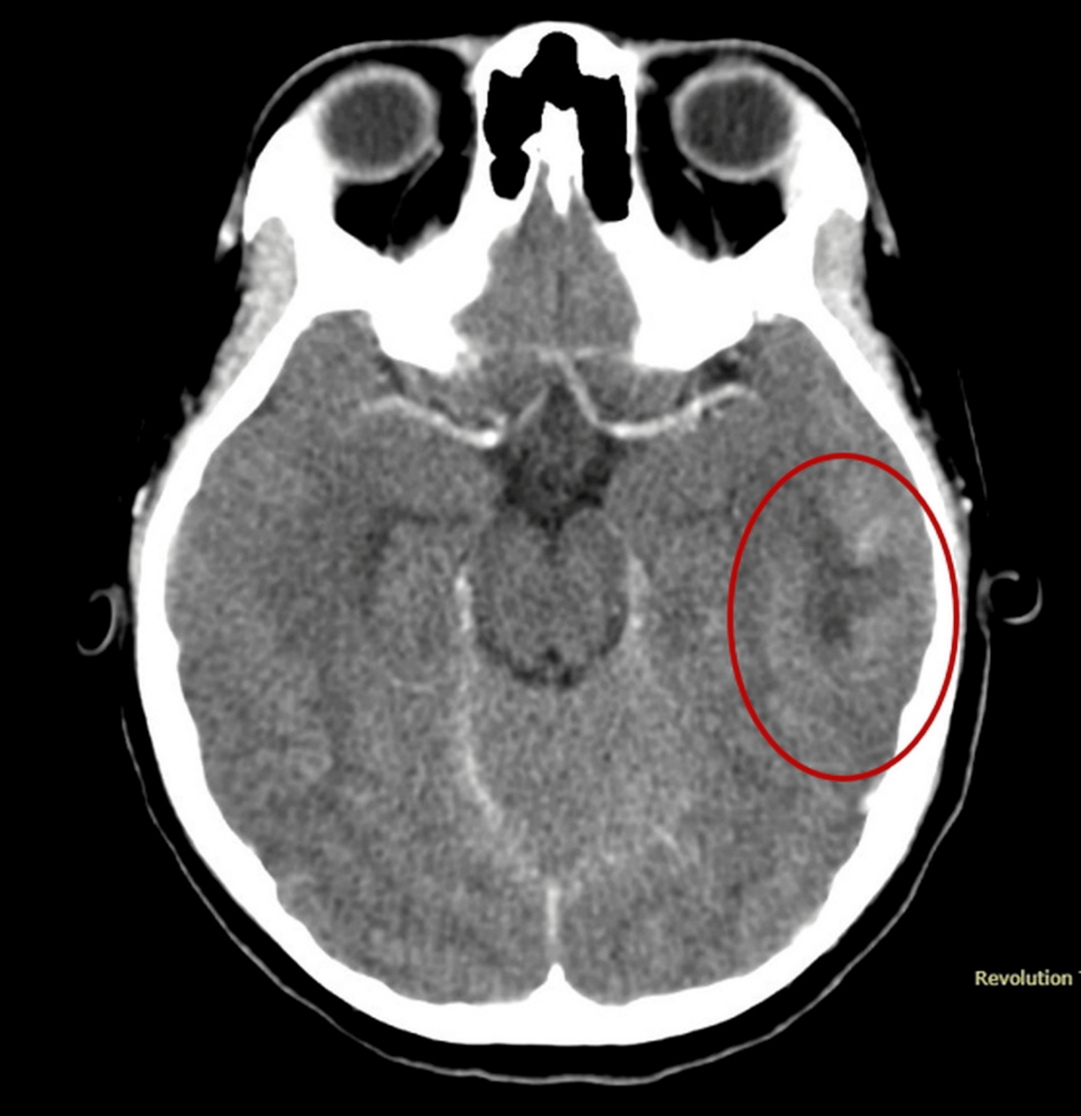
Computed tomography (CT) of the brain demonstrating cortical and subcortical infarcts in the left temporal lobe, with areas of cerebritis and small infarctions (red circle)

**Figure 2:**
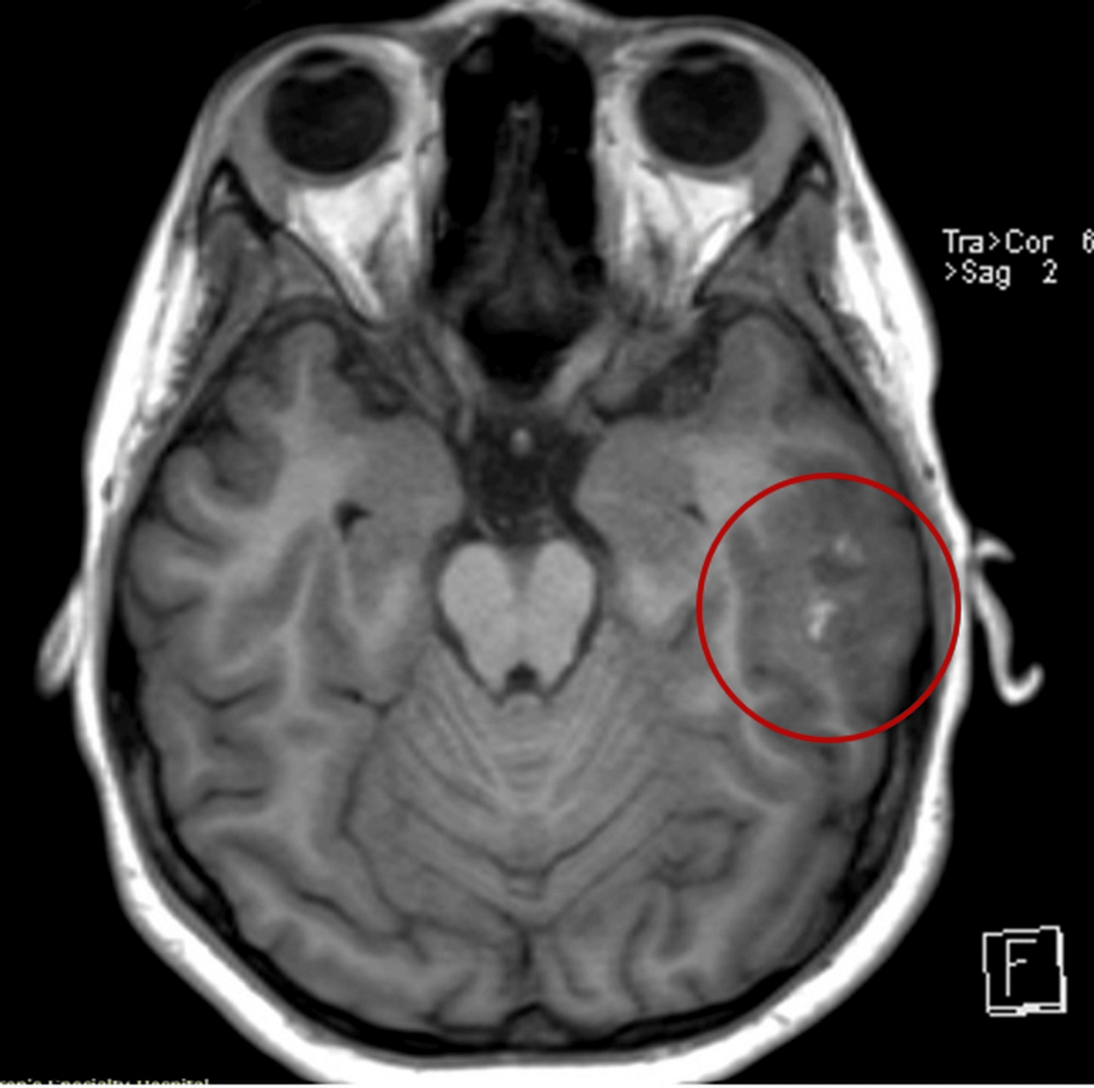
Magnetic resonance imaging (MRI) of the brain illustrating multifocal areas of cerebritis and encephalitis and associated small cavitations or abscesses within the left posterior parietal and temporal lobes (red circle)

On the third day of admission, the patient developed diplopia. Brain computed tomography (CT) and magnetic resonance imaging (MRI) revealed cortical and subcortical infarcts in the left temporal lobe, cerebritis, and small infarcts consistent with embolic phenomena (Figures 1, 2).

**Figure 3:**
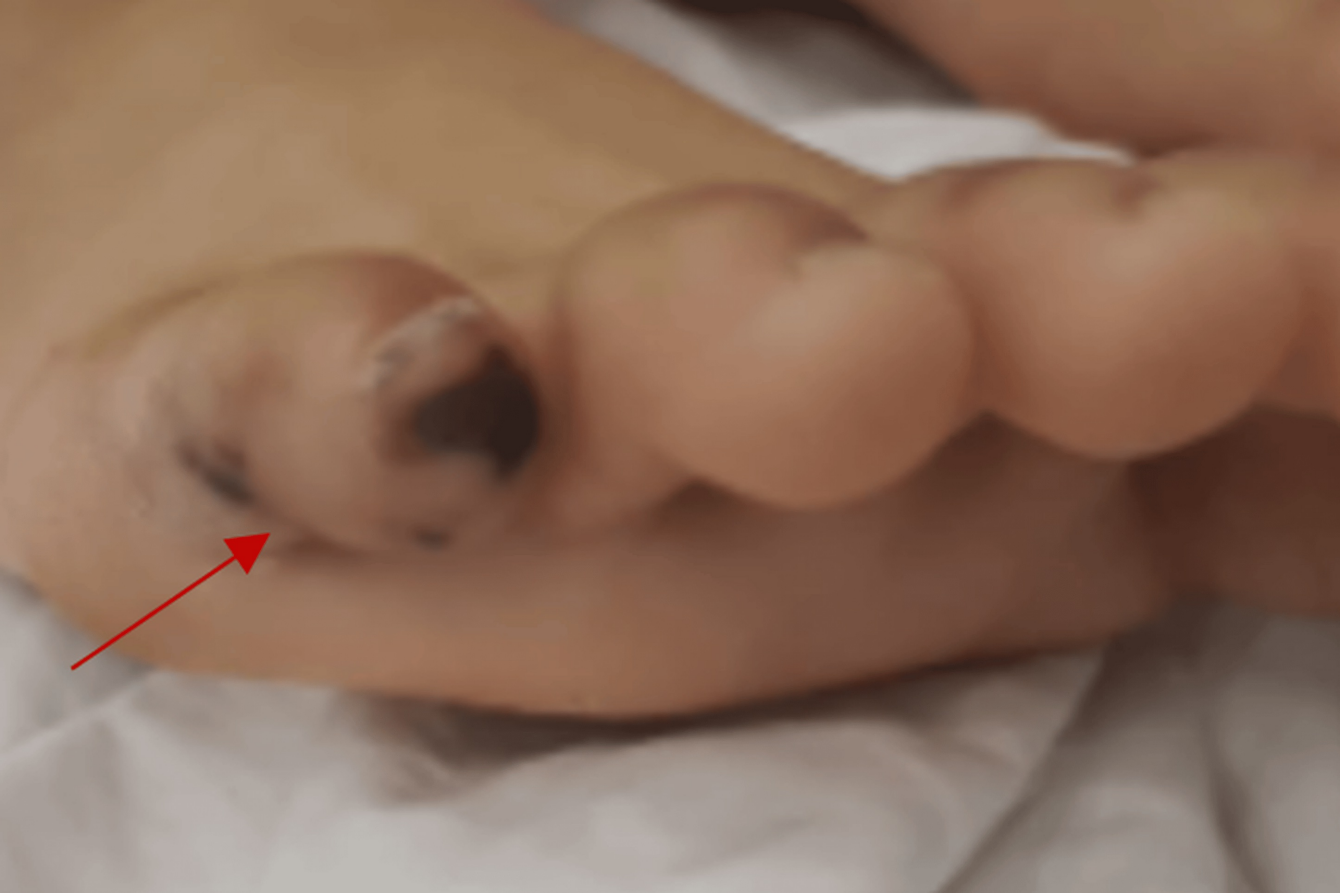
Clinical photograph showing dry gangrene of the left foot involving the little toe (red arrow)

In addition, wet gangrene was noted on the left small toe and big toe, which responded well to conservative management (Figure 3).

**Figure 4:**
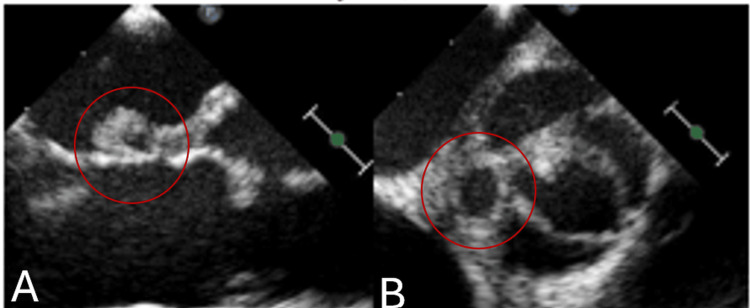
Transesophageal echocardiogram (TEE) revealing a mitral valve vegetation and an aortic sinus aneurysm with an associated abscess Parasternal long-axis and short-axis echocardiographic views, with red circles highlighting mitral valve vegetation (A) and aortic sinus aneurysm with an associated abscess (B).

TTE performed on the day of admission revealed a bicuspid aortic valve (BAV) but was otherwise nondiagnostic for vegetation. However, a repeat echocardiogram performed nine days later demonstrated a 5 mm vegetation on the anterior leaflet of the mitral valve. Serial echocardiography, conducted daily to every other day, ultimately revealed an aortic root abscess. Re-imaging also showed impaired motion of the noncoronary cusp, the presence of pseudoaneurysms, and a possible abnormal connection (Figure 4).

**Figure 5:**
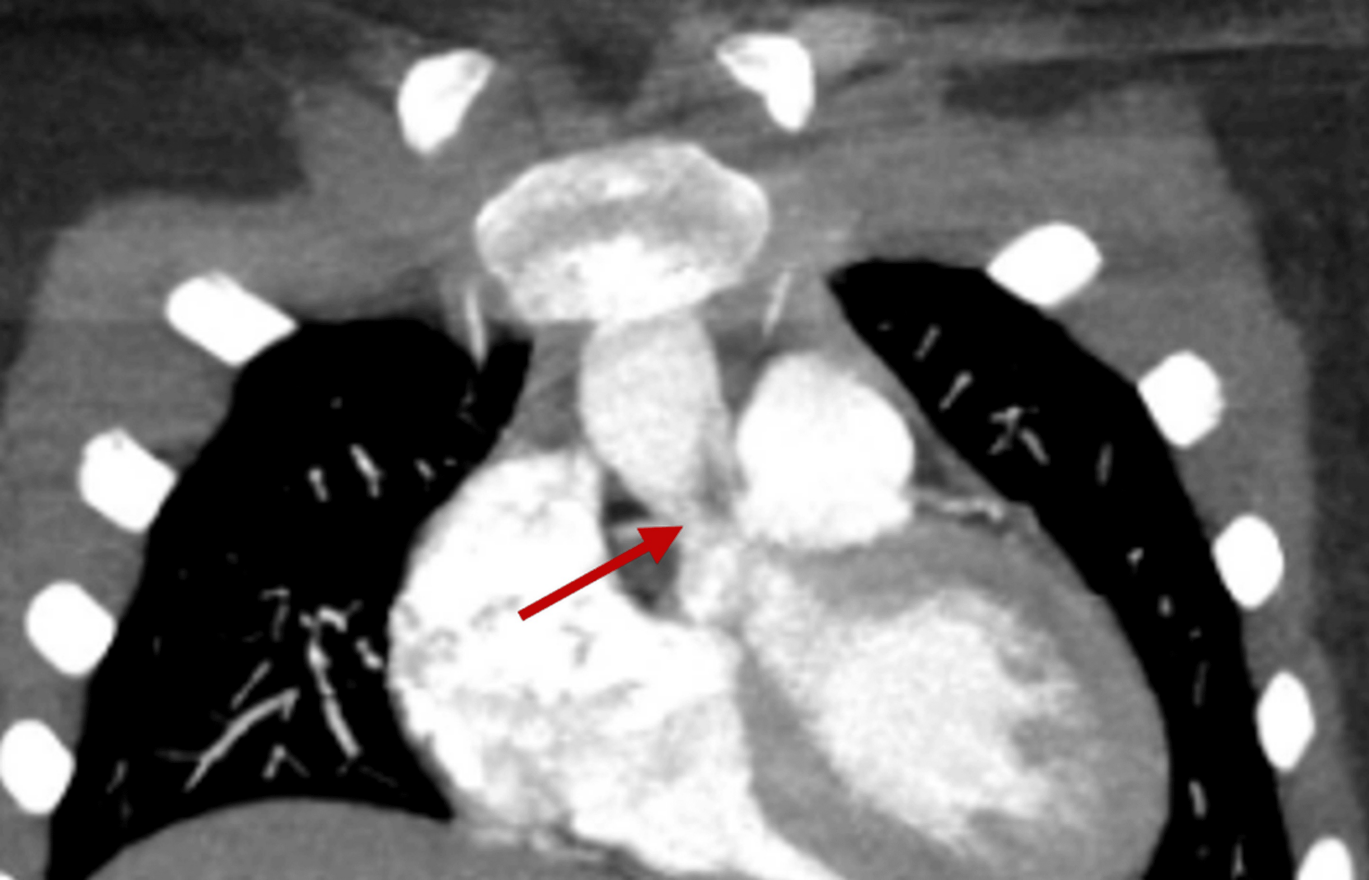
Chest CT (coronal view) showing a hypodense lesion (red arrow) at the level of the noncoronary cusp, extending to the aortic valve junction. Findings are suspicious for a peri-annular abscess

**Figure 6:**
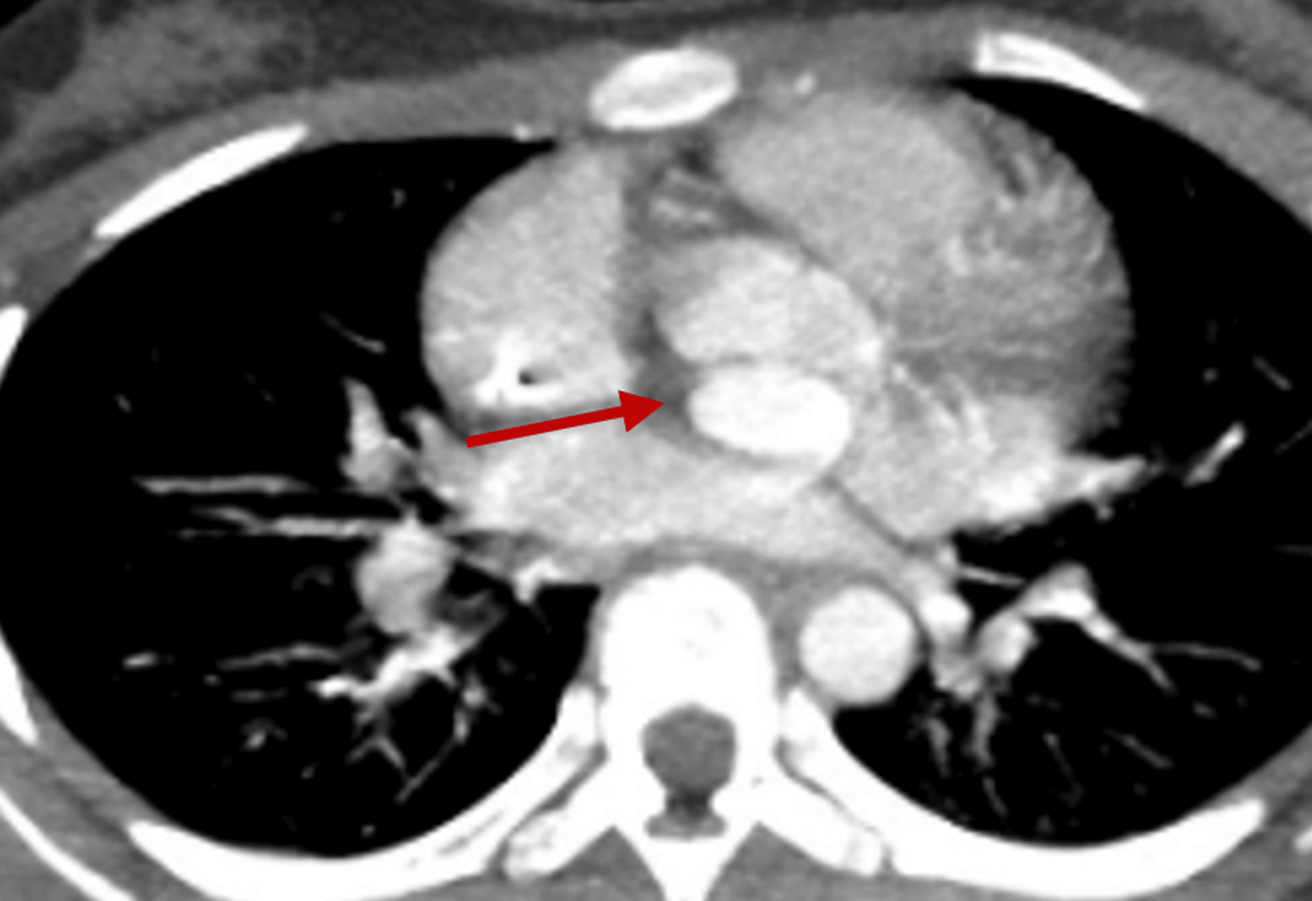
Chest CT (axial view) demonstrating the same lesion described in Figure 6, indicated by the red arrow, highlighting its spatial relationship with surrounding cardiac structures

The posterior pseudoaneurysm was filled with contrast and communicated with the posterior sinus of the aorta, showing similar contrast density to the aorta in both early and delayed imaging phases. On sagittal view, the mass measured 2.2 × 1.8 × 1.3 cm (Figures 5, 6).

**Figure 7:**
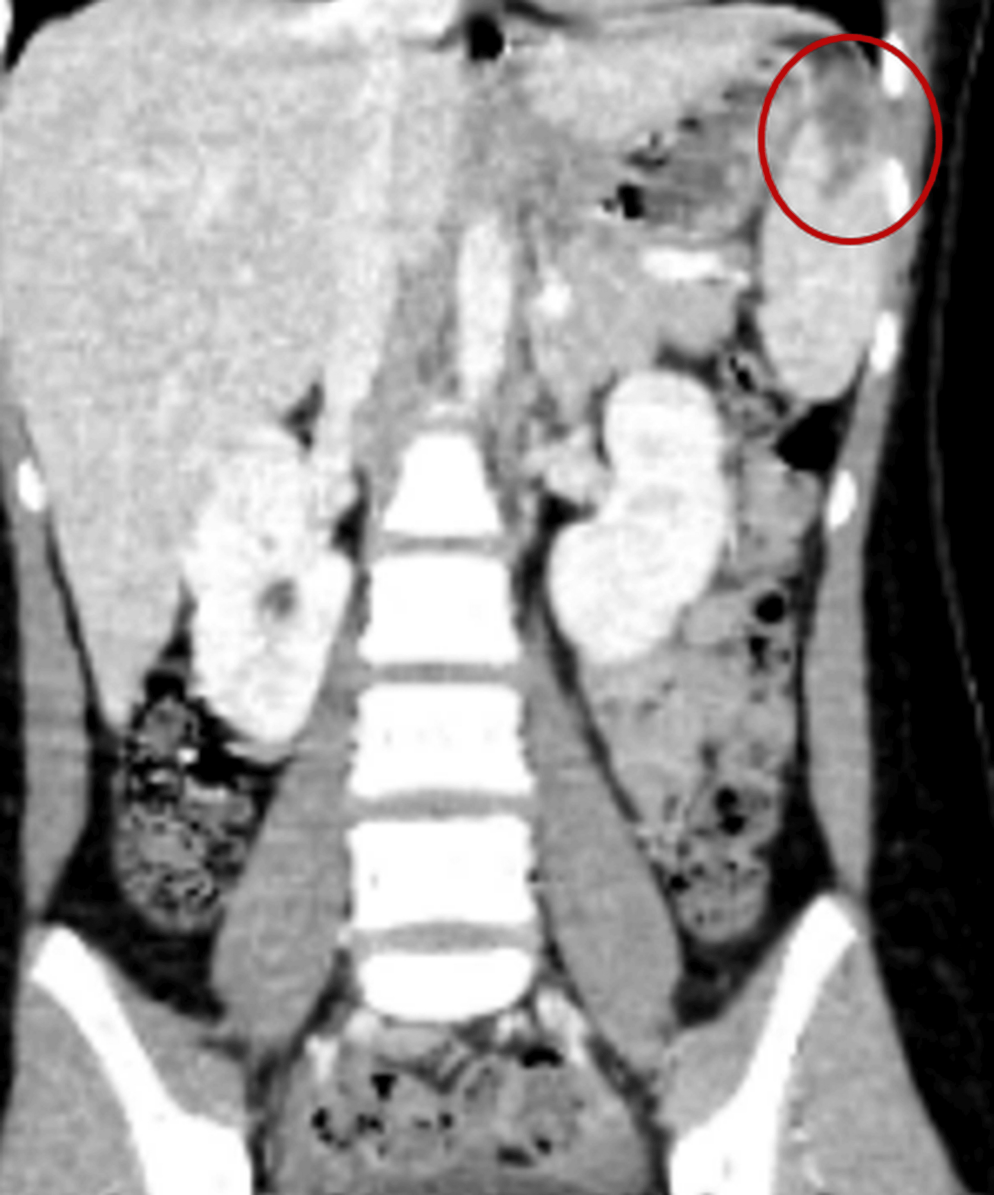
Coronal contrast-enhanced abdominal CT showing a well-defined hypodense lesion in the spleen (red circle), consistent with a splenic infarct

**Figure 8:**
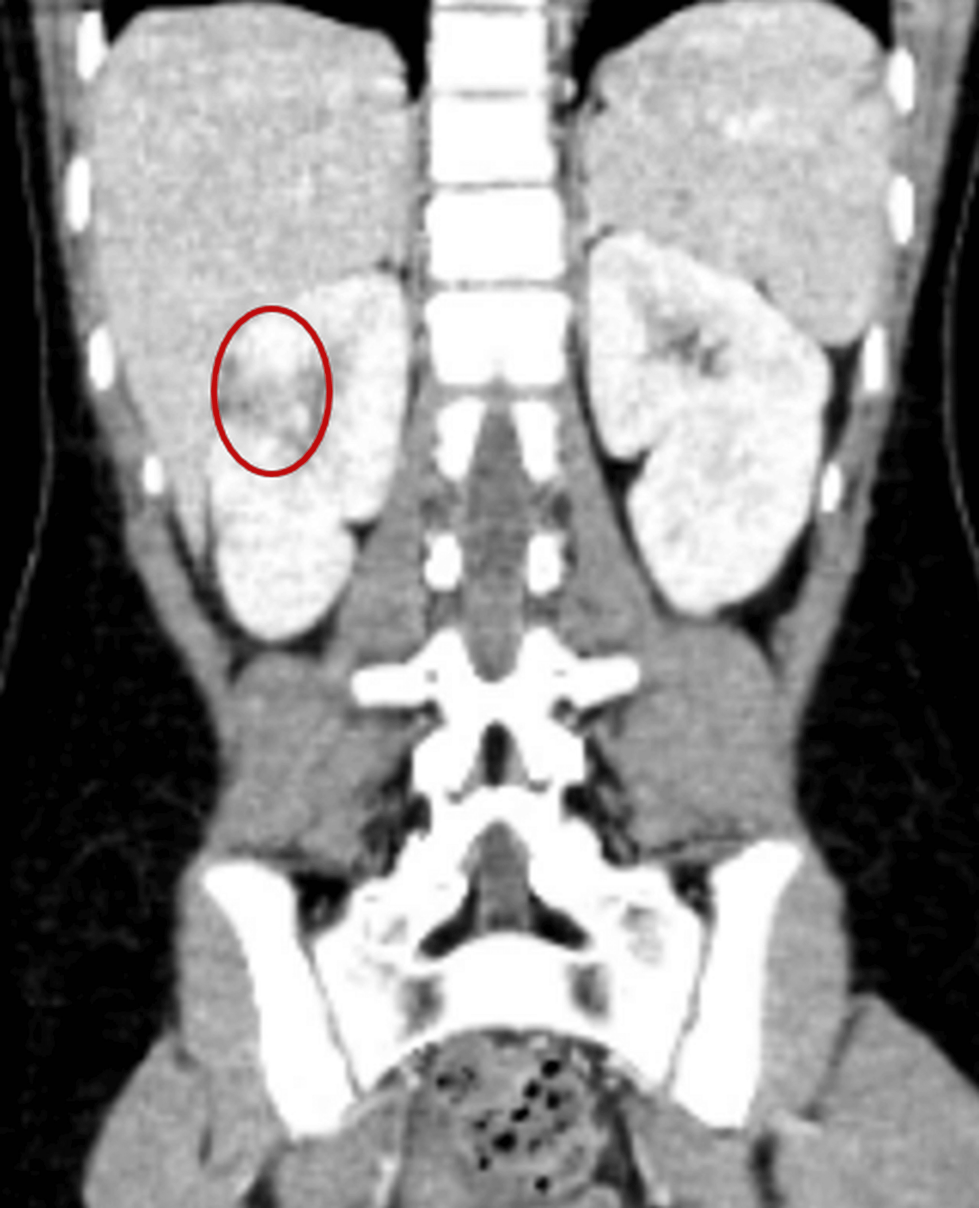
Coronal contrast-enhanced abdominal CT: right renal infarction (red circle) with a wedge-shaped hypodensity in the renal parenchyma

Abdominal and pelvic CT scans revealed splenic and renal infarctions, consistent with IE and systemic embolization (Figures 7, 8).

Course of treatment

The patient was initially started on empiric treatment for meningitis with IV ceftriaxone and vancomycin. Once MSSA was confirmed, treatment was adjusted to IV flucloxacillin. Despite appropriate antibiotic therapy, the patient’s fever persisted, and embolic manifestations, including petechiae and organ infarcts, became evident. A multidisciplinary team recommended surgical intervention based on the progression seen in follow-up imaging.

The patient underwent a modified Ross procedure. Intraoperative findings included multiple perforations of the aortic valve, an abscess cavity, and a pseudoaneurysm. The aortic valve was excised, and the abscess cavities were closed using a 15 mm autologous pericardial patch. Valve replacement was performed using the pulmonary autograft technique. Postoperatively, the patient continued on IV flucloxacillin for six weeks.

Follow-up imaging revealed resolution of abscesses and embolic lesions, with residual scarring observed in the brain, kidneys, and spleen. A whole-body MRI performed after completion of antibiotic therapy showed no new lesions or signs of active inflammation. The patient was transitioned to oral antibiotics to complete a total of six weeks of therapy post-valve replacement and was scheduled for serial outpatient follow-up, all of which have been reassuring.

## Discussion

This case highlights the diagnostic challenges of pediatric IE, particularly when early clinical features resemble more common conditions such as bacterial meningitis. The absence of specific signs in the early stages is a leading cause of delayed diagnosis, which can result in serious complications by the time IE is identified [1,4]. In our case, the initial transthoracic echocardiogram was unremarkable, underscoring the importance of repeat imaging when clinical suspicion remains high [2,4].

The management of pediatric IE should follow evidence-based protocols and require multidisciplinary coordination among pediatric infectious disease specialists, pediatric cardiologists, neurologists, and cardiothoracic surgeons [3]. The American Heart Association (AHA) recommends culture-sensitive antibiotic therapy administered for a minimum of 4-6 weeks [1]. In this patient, timely identification of MSSA enabled de-escalation from vancomycin to flucloxacillin, minimizing unnecessary exposure to broad-spectrum antibiotics and their potential adverse effects [1,3].

Echocardiographic surveillance plays a key role in detecting complications such as valvular perforation or abscess formation. In our case, serial echocardiography led to the eventual identification of mitral valve vegetation and an aortic root abscess, necessitating surgical intervention [2,4]. The modified Ross procedure, which has been supported in the literature as an effective option for pediatric IE involving both infection control and valve replacement, was successfully employed in this patient [3].

Neurological complications affect up to 40% of pediatric IE cases and are often associated with prolonged hospitalization and increased morbidity [5]. These complications include embolic strokes, cerebritis, and abscesses. Neuroimaging is essential in children with persistent fever, focal deficits, or altered mental status [5]. In this case, multiple embolic events, affecting the brain, kidneys, and spleen, as well as dry gangrene of the toes, demonstrated the extensive and unpredictable embolic burden characteristic of IE. These findings emphasize the need to evaluate for embolic complications in children who present with unexplained distal ischemia [5].

Although advances in diagnostics, surgical techniques, and antimicrobial regimens have improved outcomes in pediatric IE, long-term complications remain significant. These may include chronic valvular disease, neurologic injury, and recurrent infection [2,4]. Ongoing follow-up is crucial to monitor for late sequelae such as valve dysfunction or stenosis.

Finally, this case underscores the importance of maintaining a high index of suspicion for IE in any febrile child presenting with systemic embolic phenomena, even in the absence of known heart disease or when early diagnostic tests are inconclusive. Early recognition, serial imaging, and multidisciplinary care are critical to achieving favorable outcomes.

## Conclusions

This case underscores the importance of maintaining a high index of suspicion for IE in pediatric patients presenting with systemic or embolic-like symptoms, even in the absence of known heart disease. Early diagnosis remains challenging due to atypical clinical presentations and often negative initial imaging findings, highlighting the need for serial evaluations. Timely initiation of targeted antibiotic therapy, combined with early surgical intervention when indicated, is critical to minimizing complications and improving patient outcomes. Given the risk of neurological and embolic events, a multidisciplinary approach and ongoing long-term follow-up are essential components of effective management.
